# Gross Motor Development in Preschoolers through Conductivist and Constructivist Physical Recreational Activities: Comparative Research

**DOI:** 10.3390/sports11030061

**Published:** 2023-03-08

**Authors:** Santiago Calero-Morales, Gladis del Consuelo Vinueza-Burgos, Carlos Leonidas Yance-Carvajal, Washington Javier Paguay-Balladares

**Affiliations:** 1 Department of Human and Social Sciences, Universidad de las Fuerzas Armadas-ESPE, Quito 171103, Ecuador; 2 Faculty of Education Sciences, Universidad Estatal de Milagro, Milagro 091050, Ecuador

**Keywords:** gross motricity, physical activity, conductivism, constructivism

## Abstract

Developing gross motor function implies strengthening the basic body position and the balance associated with posture and mobility, for which different teaching models and psycho-pedagogical interventions are applied. Objective: to develop gross motor function in male preschoolers through physical recreational activities based on conductivist (Group 1) and constructivist (Group 2) teaching and determine the best teaching paradigm. Two basic skills were studied in two homogeneous independent samples (walking: w = 0.641; running: w = 0.556), selecting 25 children for each group (3–4 years) through the use of intentional sampling. The gross skills evaluation was based on norms established by the Education Ministry, including a mood assessment. Results: each group improved their basic skills in the post-test (Group 1: W = 0.001; W = 0.001. Group 2: W = 0.046; W = 0.038), but the conductivist paradigm was superior (w = 0.033; w = 0.027). Group 1 presented better indicators in the motor evaluations “Acquired” and “In Process” than Group 2, and lower percentages in the “Initiated” evaluation than Group 2 in the abilities “walking” as well as “running”, which were significantly different in the “Initiated” evaluation (*p* = 0.0469) for the walking ability, and significantly different in the “Initiated” and “Acquired” evaluations (*p* = 0.0469; *p* = 0.0341, respectively) for the running skill. Conclusions: The conductivist teaching model was superior in terms of gross motor function optimization.

## 1. Introduction

Recreational activities are understood as those that are aimed at relaxation and entertainment [1]. There are numerous types of recreation, and many of them are carried out without any delimitation of objectives, given the free nature that characterizes these activities as a sociocultural expression.

Dumazedier [2] considered recreation as a set of physical activities that are carried out once the working day is over, which can entail rest or a fun activity. He pointed out that leisure can be positive or negative depending on the kind of action since the recreational activities that are generally carried out in one’s free time do not always train a specific area. This shows the importance of investigating the differences between positive and negative leisure and proposing activities that are aimed at positive leisure, which benefits an important sector of children, namely those under 5 years of age, for which the traditional definition of recreation can set a constructivist paradigm as a fundamental form of motor teaching.

There is a large variety of activities to be carried out within the physical recreational field aimed at the integral education of children, for whom it will be easier to “learn by playing”, allowing learning to relate to the social environment [3].

To research the development of motor skills or other aspects of interest, the planning and programming of physical recreational activities are required, depending on the tastes, interests, and characteristics of the studied sample, for which prior evaluations were established [4]. These include the tastes and preferences of the sample studied to achieve motivation for physical activity and, therefore, active participation in the designed physical recreational programs. Therefore, many research papers have established strategies outside of the free choice of certain physical recreational modalities, either to favor the group or collective or to fulfill health objectives, such as obesity reduction [5,6].

Therefore, it is vital to know what types of physical recreational activities not only allow children to enjoy their free time but also serve to display some other skills necessary for their psychological, affective, and interpersonal growth, developing, among other aspects, the different basic motor skills typical of childhood [7]. In this paper, gross motor function is understood as changes in terms of body position and its ability to maintain balance, involving posture and mobility [8], as observed from the research fields of medical sciences, biology, early education, and general physical activity and health [9,10,11].

Piaget [12], one of the fathers of child psychology, affirmed that there is a close and direct relationship between physical activity and some personality facets, such as affective aspects, considering motor skills and emotions as a related whole, in which an activity is not an organism’s direct response to certain stimuli in the environment, but rather a psychomotor apparatus reaction. Therefore, physical activity influences a child’s personality development [13,14], which will be reflected in their interaction with the social environment.

Chávez and Sandoval [15] stated that the Ecuadorian children they studied have a markedly sedentary lifestyle, which influences their motor development. Durivage [16] showed that psychomotor disturbances affect body language, while Borghi [17] suggested that playing games in school is a way to control a sedentary lifestyle in a motivating way. Moreover, Parrales and Miraba [18] worked on a recreational activities guide adapted to the requirements of Ecuadorian students. Adding these studies and their themes, it is established that physical activity and recreation are necessary for a child’s motor development [19,20], with age-appropriate children’s games, and that the school must provide spaces for them for the students [21], which includes determining how to incorporate them into current teaching models in order to make them more effective.

On the other hand, when we refer to leisure and physical activity at an early age for physical and health development, we also do so by linking it to the area of pedagogy [22], which inevitably implies how it is taught and, therefore, what paradigm to use to achieve the teaching objectives. In education, specifically in the preoperative thought stage (2–7 years), various educational methods are used to engage the motor area [23], which are not unrelated to natural variations in educational content that should be used in children with or without disabilities [24].

Among the different theories of learning are behaviorism (conductivism) and constructivism. In conductivism, a teacher acts as an essential guide to the educational process [25], where the teacher is an actor with an integrated status or acts as a dynamic articulator of a complex reality. Conversely, constructivism considers that a child should “learn by doing”, being an authentic construction operated by the person who is learning, implying freedom of decisions and actions [26]. Undoubtedly, each theory has scopes [27,28] and limitations [29,30,31,32], which will depend on numerous variables, and each will create different forms of competence [33].

In the national and international literature, conductivist theories have been used in sciences, such as medicine [34], positioning it as an alternative for recovery and body strengthening, which includes learning and motor improvement, with an emphasis on people with motor limitations and various disabilities [35]. With respect to physical activity, the psychomotor point of view is of great importance to motor development, the prevention of unhealthy habits, and the promotion of establishing healthy habits, which need to be considered in order to achieve a better quality of life in the short and long term. These are the inherent and basic social responsibilities of health entities.

Knowing the optimal pedagogical strategy to promote a specific management process, such as specialized physical activity, allows for better teaching and educational results, classifying the strengths and limitations of each educational paradigm used. In this sense, it is necessary to research how to enhance gross motor function in preschoolers through physical recreational activities, assessing whether the conductivist or constructivist educational model best enhances them in the two independent study samples.

## 2. Methods

### 2.1. Participants

The research studied 50 subjects classified into two independent samples made up of 25 male children in initial education (3–4 years old), using a conductivist educational model (Group 1) and another constructivist model (Group 2). The children investigated attend Escuela de Educación Básica Fiscal San Felipe Neri in Quito, and the selection of the children was based on an intentional non-probabilistic sampling, which avoids effects that different teachers may have in the learning/teaching process on psychomotor skills, while the participating teachers remained throughout the intervention process as a control for possible reactive-type methodological effects, avoiding possible variations in the experimenter/observer effect that could cause variations in the reliability of the study.

The inclusion criteria of the children were: (a) a high participation in the intervention proposal (≥92%; dropout rate: 0%), (b) presenting low gross motor performance in the lower limbs, (c) not presenting motor disability/intellectual or previously diagnosed developmental disorders, (d) not presenting physical injury during the physical recreational intervention that might cause abandonment of class sessions or significant non-attendance, (e) being male and in the mentioned age range, (f) and that each independent group had a similar index of gross motor quality in the walking and running ability (w = 0.641; w = 0.556, respectively) before starting the intervention process (pre-test).

### 2.2. Instruments

Data on the preferences and motor influence in physical recreational activities proposed in the “Procedure” section were obtained, verifying the child’s motor–physical development, performance, abilities and shortcomings when complying with them, using an experimental model as a manipulative strategy [36]. For this, exercises were carried out by 25 male children in terms of running and walking, in which the teacher guided the entire process rigidly (Group 1), and by another 25 male children of the same age to whom the same physical recreational proposal was applied based on a constructivist educational model (Group 2), where the teacher allowed total freedom of action/decision to the children under study.

The assessment levels of the activities carried out have been established according to the 2014 Initial Education curriculum of the Republic of Ecuador [37], in which the skills are qualified as follows: Acquired (3 points), In Process (2 points), and Initiated (1 point); adapting the TEPSI test scores [38] in the motor component for walking ability and the TGMD 2 [39] for running ability, and classifying the following indicators related to the gross motor skills to be evaluated:

(1) TEPSI (walking ability). The execution criteria:
  (a) Walk forward less than four steps in a straight line and without support, touching the heel with the toe (Initiated).
  (b) Walk forward between four to six steps in a straight line and without support, touching the heel with the toe (In Process).
  (c) Walk forward more than six steps in a straight line and without support, touching the heel with the toe (Acquired).
(2) TGMD 2 (running ability): The execution criteria:
  (a) Arms move in opposition to legs, elbows bent (Initiated).
  (b) Brief period where both feet are off the ground (Initiated).
  (c) Narrow foot placement landing on heel or toe, not flat-footed (In Process).
  (d) Non-supported leg bent approximately 90° degrees, close to buttocks (Acquired).

On the other hand, to determine the mood state of the studied samples, direct questions were asked by the teacher (whether they liked the activity or not). The mood state was determined at the end of the physical recreational activity in the final part (farewell), in a random way, performing for control in the last three weeks of the intervention program. The mood state indicator was determined by the child’s body–facial expression and classified as either excellent, good, or normal, as these are innate and phylogenetically determined behaviors [40].

### 2.3. Procedure

One of the first steps was obtaining the necessary permissions from the school authorities to carry out the research, subsequently informing parents/teachers of the objectives of the intervention process and its theoretical justification, including advantages and research limitations, as well as the instruments used. The parents/teachers authorized the children’s participation in the research voluntarily and anonymously.

In the research implementation, international ethical guidelines were adopted, emphasizing guideline 17, as prepared by the Council for International Organizations of Medical Sciences (CIOMS) in collaboration with the World Health Organization (WHO), in relation to the right to self-determination of the subjects studied. The parents/teachers previously provided informed consent, which included the assent of the children studied, in compliance with the guidelines approved in the Declaration of Helsinki.

The research guaranteed homogeneity in the groups before the intervention started by comparing their initial gross motor skills, which were found to be similar in both independent groups, as specified in the number inclusion criterion. The data collection was carried out in two stages (pre-test and post-test), analyzing under all times conditions, such as illness, excitement, and accidents, among others, as established by Symonds [41], to guarantee that the control tests were carried out under identical objective conditions in order to achieve reliability and less variability in the indicators that intervene directly and indirectly in the performance assessment tests (the subjects’ distribution by independent groups and classes types, environmental and space situations, intervention schedule, the teaching materials, and the equipment used in each class, the recollection, recording and methodology used), ensuring that the analysis procedure did not affect the research results.

The children were randomly assigned to each independent group by the school director, with three teachers participating in the research, one for each intervention group, and the third interspersed, supporting each group randomly and checking that the pedagogical guidelines were met. All of the teachers who participated in the intervention had extensive teaching experience (≥15 years). Additionally, three independent evaluators were used, recording and processing the basic motor skills evaluated at the two control moments. Evaluator 1 was classified as “expert”, and the remaining two were used to check coincidences in the expert’s evaluation, in which Krippendorff’s alpha was taken as evidence of agreement between the observers.

It was ensured that all the children in both independent groups would present a similar level of gross motor development before implementing the proposed physical recreational activities for six months (duration: 35 min in its principal part; frequency: 3 sessions a week). Activities were oriented towards motor play, motor song, motor story, and motor circuits, according to didactic and methodological considerations of the child’s motor skills [42]. The evaluation of the independent groups conformed with the norms established for initial education provided by the Education Ministry [37].

Regarding the methodology and content of the children’s physical preparation to enhance their gross motor skills, it was taken into account as part of the physical education class to prioritize the following physical recreational actions:

The physical recreational activities to enhance gross motor function were basically designed as follows:

#### 2.3.1. Walking

Coordinated Walking: Perform Coordinated Walks Following the Commands: Walk like a Soldier, Walk like the President, Walk like an Ogre, and Walk like a Mouse.

Walking Keeping Your Balance: Carry out a Race on the Edge of the Institution’s Curbs.

Walking at Different Rhythms: Recreational Activities: Develop a Modelling Contest with Music of Different Types.

#### 2.3.2. Running

Recreational Activities Level 1: Carry out a Race with Different Obstacles Such as Hula Hoops, Cones and Ropes.

Recreational Activities Level 2: Carry out Relay Races. The Children Must Be Divided into Two Groups, One Child Must Wait at One End, the Partner Brings the Ball and from There the First Child Runs to Hit the Next Partner.

Recreational Activities Level 3: Play the Popular Game of Cat and Mouse, and Other Alternatives.

The aforementioned physical recreational actions were a priority in the principal part of the class (35 min), with the basic walking and running skills prevailing; for these, the specialization principle was emphasized as determinant abilities of human locomotion at the expense of other basic physical abilities that were considered in the research as complementary since they were not taken into account in their control, such as turning, catching, throwing, hitting, and jumping.

The methodological characteristics applied in Group 2 (Constructivism) included a regular physical activity program normally used in schools, including the Escuela de Educación Básica Fiscal “San Felipe Neri” in Quito, from which the study sample was drawn. The basic methodological characteristics are described below:

(1) The intervention program objective: to improve gross motor skills by strengthening two basic motor skills (running and walking).
(2) The non-obligatory verbal guidance to randomly carry out the physical recreational actions mentioned above, without an emphasis on the systematic correction of the motor error, and without the intensive use of the explanatory–demonstrative method by the teacher.
(3) Free play was tolerated if the child consciously decided to change the physical content proposed. There was teacher supervision of all physical activity presented in the principal part of each class, carried out in the school playground or gym, with the equipment normally used in such activities (balls, hoops, bats, cones, ropes, chairs, etc.).
(4) The professional development of the participating teacher did not include systematic training beyond the basic guidelines since constructivist free play was part of the school’s regular physical activity program.

The basic methodological characteristics applied in the physical recreational actions for Group 1 (Conductivism) are described below:

(1) The intervention program objective: To improve gross motor skills by strengthening two basic motor skills (running and walking).
(2) The teachers participating in group 1 underwent a course endorsed by the Department of Human and Social Sciences of the Universidad de las Fuerzas Armadas-ESPE (32 contact hours), with psychomotor aspects based on the model “Educa a tú Hijo/Educate your Son” for children aged 3–5 years old [43], an internationally and methodologically endorsed program, characterized by the joint working of the family and the community (including the teaching area), which includes the structured direction of physical stimuli, such as those present in Roa-González et al. [44], with an eminently conductivist management, where teachers are fundamental actors in the learning–teaching process.
(3) The systematic implementation of the aforementioned physical recreational actions, concentrating physical activities to develop the basic skills of running and walking as a basis for enhancing gross motor skills. Increase and additional adaptations of the physical recreational actions were accepted, provided they were directly related to the research purpose and the physical recreational actions described.
(4) Systematic correction of errors and an intensive use of the explanatory–demonstrative method by the teacher.

The intervention process in both independent groups includes in each class a structuring by time that contains an initial part (communication: where the teacher guides the class objective and exchanges comments, feelings and experiences) and a final part (farewell: where the teacher assesses the class motivation, allows the children to recover, and talks about the class achievements), each lasting 5 min, and the principal part lasting 35 min (total: 45 min), where he introduces the intervention mechanisms according to the class objective. In general, it was checked that no independent group that participated during the research time was taking part in any other organized program of physical activity.

As the conductivist and constructivist teaching models are commonly used in various teaching–learning processes, they are not considered to present any considerable risk from the viewpoint of physical and psychological health, complying with the requirements of the Ethics Committee of the Universidad de las Fuerzas Armadas-ESPE (ID: 2016-104-ESPE-d).

### 2.4. Data Analysis

To compare the basic skills level of each group studied, the proportions for the independent samples’ “CPMI” (*p* ≤ 0.05) were calculated, evaluating the absolute and percentage frequencies of each motor achievement, reinforced with the non-parametric statistic for the two independent samples’ Mann–Whitney U test (w ≤ 0.05), applied at two intervention moments (pre-test and post-test), since there was no normal distribution data according to the Shapiro–Wilk test. The first application of the Mann–Whitney test (pre-test) was used as a homogeneity indicator for the two independent samples studied. Prior to this, the Wilcoxon Signed-Rank Test (W ≤ 0.05) was used to assess the results for the two related samples, comparing the gross motor performance of the independent groups with each other and assessing whether there were improvements in basic skills in either of the two educational paradigms.

For the motor skills evaluation, three independent observers with previous training in the acquisition of conceptual maturity were used. The internal observational reliability achieved a “Satisfactory Agreement” in the running (α = 0.8401) and the walking ability (α = 0.8125) as part of the post-test, according to Krippendorff’s alpha, an aspect that evidenced agreement between the observers, and therefore, the reliability of the evaluative records. To analyze all of the data, the SPSS v.25 statistical package was used, with the exception of the “CPMI” calculation, for which the Statistics for Windows v.5 software was used.

## 3. Results

Table 1 shows improvements in gross motor skills in both independent groups, where Group 1 presented, according to the Wilcoxon Test, favorable differences in the post-test in the walking ability (W = 0.001) and running ability (W = 0.001), while in Group 2, the differences were also significant in the post-test in terms of walking (W = 0.046) and running (W = 0.038). This shows that regardless of the conductivist and constructivist paradigm applied, each independent group improved their gross motor skills.

However, when comparing the results as independent samples, Table 2 shows the nonexistence of significant differences in the previous evaluations (pre-test) for the intervention process in each motor skill evaluated (walking: w = 0.641; running; w = 0.556), which is indicative of a similar performance in gross motor skills in each independent group, a homogeneous performance that must be controlled as an external variable to avoid falsifying the data in its final process. On the other hand, the results obtained as part of the post-test showed significant differences in favor of Group 1 (conductivism), with notable differences in terms of walking ability (w = 0.033) and running ability (w = 0.027), given the average ranges analysis (AR), and these were in all cases greater than those obtained by Group 2 (constructivism).

Table 3 shows a more detailed analysis of the absolute and percentage frequencies achieved in each qualitative result in the post-test part after completing the intervention.

A final diagnosis of walking and running ability was made in both groups studied after implementing the physical recreational actions designed (Table 3). In the walking skill, 48% of children did not walk correctly (Group 1), while 76% (Group 2) had the same problem after implementing physical recreational activities. The groups presented deficiencies, with the highest percentage placed at the skill Initiated level, followed by the In Process level (Group 1: 44% and Group 2: 24%). The most common difficulties were lacking balance, and among the frequent errors verified were not looking forward, not coordinating the arm and leg movements, and not holding the head and trunk upright.

As for the running ability, 48% of the children performed a regular run, equivalent to a higher percentage for Initiated skill than the rest, with 20% In Process, while only 32% were rated excellent, with an evaluated result of Acquired. Regarding the running ability for Group 2, 76% of the children were assessed with an Initiated evaluation, 16% In Process and 8% Acquired. For both groups, the most common errors detected were not looking straight ahead and the trunk leaning slightly forward.

Significant differences were detected in the walking ability (Initiated evaluation) between the independent groups studied (*p* = 0.0469), according to CPMI, with Group 1 having the lowest percentage of students evaluated with the worst rank in the final motor control tests (Group 1: 48%; Group 2: 76%), while Group 1 presented a higher percentage, although not significant, in the ranges evaluated, with a better indicator of motor development for the walking ability (In Process: 44%, Acquired: 8%) with respect to Group 2, where a teaching model based on constructivism was implemented (In Process: 24%, Acquired: 0%).

In the running skill case, the research determined that Group 1, where the gross motor function was improved through a teaching model based on conductivism, was the one that presented the best indicators, with the value being significantly different in the “Initiated” evaluation (*p* = 0.0469), indicating a lower percentage of children with the worst evaluations in a fundamental motor skill (Group 1: 48%, Group 2: 76%), while in the “Acquired” evaluation also Group 1 presented the best percentages, the comparison between groups being significantly different (*p* = 0.0341). Therefore, Group 1 presented a higher percentage of children with excellent evaluations for the study age.

For both groups, the mood was determined as excellent in most cases (Group 1: 80%, Group 2: 88%), as evidenced in Table 4, without presenting significant differences in any compared case (excellent: *p* = 0.4442; good: *p* = 0.6395; normal: *p* = 0.5543), evidencing a positive mood during the activity, as reflected in smiles and pleasure in participating in any physical recreational activity, whether this was a physical activity structured under the conductivist paradigm or a freer physical activity managed under the constructivist paradigm.

## 4. Discussion

The research determined that the children studied had difficulty with basic motor skills at the beginning of the investigation, from which it was deduced that there is little attention from the parents and teachers to early stimulation, an aspect that seems to be common in the Ecuadorian environment according to Cabrera’s indications [45], and that requires the improved use of leisure with active physical activities according to Sandoval-Jaramillo et al. [46], given that nowadays, technological activities tend to be promoted more in free time (computer, tablet, and television) than cooperative and family physical play, which promotes a sedentary lifestyle and associated psychomotor problems [2,47]. Believing that children are physically active all the time is a very common error. Physical activity levels in children can be influenced by various factors, including the recent COVID-19 pandemic, as evidenced by Neville et al. [48], which can be markedly unfavorable in low-resource areas, as established by Lee et al. [49].

In general, among the most common errors when walking was a lack of balance. This difficulty may be due to psychomotor disorders, as considered by Durivage [16] when he states that psychomotor disorders are delays or difficulties that arise during walking psychomotor evolution, and it manifests itself through clumsy movements, stiffness, lack of balance or tonic control, aspects that tend to be exacerbated nowadays given the technological habits acquired at home by new generations [50], mostly due to a deficit of moderate activity and systematic physical activity. This requires insistence and active management by teachers to recover the physical habits, for which the conductivist model could be ideal in certain age ranges.

Common or acquired errors in skills such as running or walking, at least at an early age (3–4 years), must be continually corrected with specialized advice and family support [43] since it is precisely at school and in the family where a child spends a considerable time in their life and acquires certain sociocultural behaviors. In this sense, a conductivist teaching model tends to be more appropriate for enhancing basic motor skills since this educational model prioritizes reinforcing concrete actions that optimize specific motor movement processes at the study age. Therefore, in the present research, the approaches proposed by Kirschner et al. [31] are reinforced, in that beginners do not have the underlying mental models or schemes necessary to “learn by doing” or, as Mayer affirms [30], learners must be cognitively active in study, but teachers must employ directed practice. In this sense, a constructivist model, in the case of physical activity and health, could eliminate objective criticism and professional debate, in addition to making teachers dispensable [29], as evidenced by practice.

Authors such as Goodway and Branta [51] show in their paper that the lack of an organized approach to game components causes insufficient increases in the motor percentile in preschool children (0.4%). This lack of an organized approach is evident in the constructivist teaching paradigm, given the freedom of choice that the child has to carry out physical activities, selecting on numerous occasions passive activities that do not enhance locomotion. In addition, Miller [52] determined that direct instruction to children improves their performance to a greater extent than children participating in a well-equipped playgroup. In the present research, such direct instruction, typical of conductivism, allowed a notable improvement in walking and running skills, respectively (w = 0.033; w = 0.027).

An interesting paper related to the present research was published by Ruiz-Esteban et al. [53], who show that motor skills instruction in a directed program has a positive impact that exceeds a free play program in terms of the development of fundamental motor skills in preschool, although their research applied two totally different programs of physical intervention, one for each independent sample. The aforementioned authors’ results are supported by other earlier research, where it is specified that sessions structured through a functional and directive intervention methodology for children 3–5 years old present better results in motor performance than an intervention model or experiential and non-directive exercise [54,55]. However, although free physical activity based on a non-directive experiential physical model may be indirectly related to the objective of this research, the purpose of this report is not to compare two indistinct models of physical recreational intervention but rather to compare how it manifests the same physical recreational intervention model applying different educational paradigms, although the constructivist paradigm significantly modifies its previously structured contents through the child’s practical attitude by implementing a “learning by doing” or “learning by playing” approach.

In the present case study, both the constructivist and conductivist models showed a positive mood state when engaging in physical recreational activities, with the constructivist model showing the highest percentage from the mental viewpoint (Constructivism: 88%; Conductivism: 80%), although this was not expressed in a significant way (*p* = 0.4442). The foregoing implies that motor play is a natural expression at the age studied and that the simple fact of participating physically and recreationally with the group implies a high degree of motivation in the child [56] although, according to the research conclusions of Andrade et al. [57], creating a task-oriented environment during classes encourages more active behaviors, as managed in conductivism.

Although the child can be further motivated by performing free physical recreational activities, children currently tend to select passive recreational activities (games without considerable motor movement), many of which are chaotic, limiting continuous training and the systematic correction of motor errors, aspects that are normally intensively managed in the conductivist model. Given the above, the constructivist model improved gross motor skills but did not optimize the motor function in the present investigation, requiring the teacher’s direct intervention as an essential guide in the educational process, dynamically integrating and articulating reality, as established in conductivism [25]. However, the student’s autonomy should not be ruled out in an integral teaching/educational model but is a fundamental component of free physical activities that allows the full development of motor skills [58] and therefore is usually a determining component of motor performance, normally managed in a constructivist paradigm.

In terms of practical applications, the research results will make it possible to theoretically and methodologically establish the most optimal teaching paradigm to develop gross motor skills in children from physical education, an extremely useful aspect to enhance sports performance as part of the search process and sports selection from early ages, sports performance that depends on the development of basic physical skills, and therefore, the gross motor development level.

### Strengths and Limitations

Considering the characteristics and results evidenced in the research, caution should be exercised given the non-representative sample size and considering that the male gender results may differ from the female gender not studied here, in addition to taking into account that children’s performance in the age range studied can vary in other stages of human motor development. There is a need to carry out larger cross-sectional research, which includes motor assessment tests applied to an educational context of better psychometric quality, as stated by Ruiz-Esteban et al. [53], emphasizing the children’s mood at 3–5 years old during the performance of specialized physical activities. In addition, given the limitation of evaluating only two basic physical skills that are essential to human locomotion (walking and running), in the future, it will be necessary to consider the integrated nature of various motor skills not considered in this research, which could present some peculiar variation or characteristic that modifies the data obtained.

The basic strength is the originality of the research since no empirical research directly applied to the study field for developing gross motor skills through the educational paradigms analyzed was found in the literature. The analyzed data can serve as a starting point for further research that theoretically and methodologically supports the choice of free play and structured play to optimally develop gross motor skills based on the optimal pedagogical paradigm to be used, the present research being a pertinent and original study, and a pioneering example.

## 5. Conclusions

The educational paradigms studied improve motor skills based on walking ability (conductivism: W = 0.001; constructivism: W = 0.046) and running ability (conductivism: W = 0.001; constructivism: W = 0.038), but the conductivist paradigm was better for completing the research objective, as evidenced in the post-test (walking: w = 0.033; running: w = 0.027), given the existence of a higher average range in terms of walking ability (29.24AR) and running ability (29.44 RA). Physical recreational activities should be carried out in a planned, conscious, systematic, structured, and professionally directed manner to better enhance gross motor function in male children aged between 3 and 5 years, fundamentally based on a conductivist paradigm that should limit the difficulties as an educational model. Furthermore, it is recommended to permit a certain degree of flexibility in the programmed application, promoting motivation and differentiated physical loads according to the needs, possibilities, and sample characteristics, thus promoting certain advantages of the constructivist model for the age researched here.

## Figures and Tables

**Table 1:** Wilcoxon Signed-Rank Test.

		Ranks			
			N	Mean Rank	Sum of Ranks
	Negative		0 ^a^	0.00	0.00
walk.Group1.Postest-walk.Group1.Pretest	Positive ranks		11 ^b^	6.00	66.00
	Ties		14 ^c^		
	Total		25		
	Regative ranks		0 ^d^	0.00	0.00
run.Group1.Postest-run.Group1.Pretest	Positive ranks		13 ^e^	7.00	91.00
	Ties		12 ^f^		
	Total		25		
	Regative ranks		0 ^g^	0.00	0.00
walk.Group2.Postest-walk.Group2.Pretest	Positive ranks		4 ^h^	2.50	10.00
	Ties		21 ^i^		
	Total		25		
	Regative ranks		0 ^j^	0.00	0.00
run.Group2.Postest-run.Group2.Pretest	Positive ranks		5 ^k^	3.00	15.00
	Ties		20 ^l^		
	Total		25		
Test Statistics					
walk.Group1.P ostest-walk.Group1.P retest		run.Group1.Po stest-run.Group1.Pre test		walk.Group2.P ostest-walk.Group2.P retest	run.Group2.Po stest-run.Group2.Pre test
Z	−3.207 ^m^	−3.272 ^m^		−2.000 ^m^	−2.070 ^m^
Asymp. Sig. (2-tailed)	0.001	0.001		0.046	0.038
	a. Based on negative ranks.				
	m. Wilcoxon Signed-Rank Test.				

^a^: walk.Group1.Postest < walk.Group1.Pretest; ^b^: walk.Group1.Postest > walk.Group1.Pretest; ^c^: walk.Group1.Postest = walk.Group1.Pretest; ^d^: run.Group1.Postest < run.Group1.Pretest; ^e^: run.Group1.Postest > run.Group1.Pretest; ^f^: run.Group1.Postest = run.Group1.Pretest; ^g^: walk.Group2.Postest < walk.Group2.Pretest; ^h^: walk.Group2.Postest > walk.Group2.Pretest; ^i^: walk.Group2.Postest = walk.Group2.Pretest; ^j^: run.Group2.Postest < run.Group2.Pretest; ^k^: run.Group2.Postest > run.Group2.Pretest; ^l^: run.Group2.Postest > run.Group2.Pretest; ^m^: run.Group2.Postest = run.Group2.Pretest.

**Table 2:** Mann–Whitney U test.

Rangos				
	Group	N	Mean Rank	Sum of Ranks
walk.Pretest	Conductivism	25	26.00	650.00
	Constructivism	25	25.00	625.00
	Total	50		
walk.Postest	Conductivism	25	29.24	731.00
	Constructivism	25	21.76	544.00
	Total	50		
run.Pretest	Conductivism	25	26.00	650.00
	Constructivism	25	25.00	625.00
	Total	50		
run.Postest	Conductivism	25	29.44	736.00
	Constructivism	25	21.56	539.00
	Total	50		
**Test Statistics ^a^**				
	walk.Pretest	walk.Postest	run.Pretest	run.Postest
Mann–Whitney U	300.000	219.000	300.000	214.000
Wilcoxon W	625.000	544.000	625,000	539.000
Z	−0.467	−2.134	−0.590	−2.210
Asymp. Sig. (2-tailed)	0.641	0.033	0.556	0.027

^a^. Grouping Variable: Group.

**Table 3:** Walking and running ability: final diagnosis.

			Walk			
Result	Group 1. Conductivism	%	CPMI	Result	Group 2. Constructivism	%
Acquired	2	8%	0.1554	Acquired	0	0.00%
In process	11	44%	0.1421	In process	6	24%
Initiated	12	48%	0.0469	Initiated	19	76%
Totals	25	100%		Totals	25	100%
			**Run**			
Acquired	8	32%	0.0341	Acquired	2	8%
In process	5	20%	0.7144	In process	4	16%
Initiated	12	48%	0.0469	Initiated	19	76%
Totals	25	100%		Totals	25	100%

CPMI: Calculation of proportions for independent samples.

**Table 4:** Mood state when carrying out the physical recreational activity.

Result	Group 1. Conductivism	%	CPMI	Result	Group 2. Constructivism	%
Excellent	20	80%	0.4442	Excellent	22	88%
Good	3	12%	0.6395	Good	2	8%
Normal	2	8%	0.5543	Normal	1	4%
Totals	25	100%		Totals	25	100%

## Data Availability

The data presented in this study are available on request from the corresponding author. The data are not publicly available due to the qualitative nature of the study.
